# 3D‐Printed Cosmetic Enhancements Guided by Artificial Intelligence

**DOI:** 10.1111/jocd.70263

**Published:** 2025-05-27

**Authors:** Marina Landau, Maria Tsoukas, Mohamad Goldust

**Affiliations:** ^1^ Arena Dermatology and Department of Plastic Surgery Shamir Medical Center Be'er Ya'akov Israel; ^2^ Department of Dermatology University of Illinois College of Medicine at Chicago Chicago Illinois USA; ^3^ Department of Dermatology Yale University School of Medicine New Haven Connecticut USA

**Keywords:** 3D printing, 3D‐printed cosmetic procedure, artificial intelligence, dermatologic surgery

Dear Editor,

Artificial intelligence (AI) and 3D printing are coming together to transform aesthetic dermatology through personalized, data‐driven cosmetic interventions. By integrating facial scans, dermatologic imaging, and predictive algorithms, AI enables the development of patient‐specific implants and fillers with remarkable precision. This collaboration creates new opportunities for delivering minimally invasive, efficient, and predictable aesthetic enhancements [1, 2, 3].

One significant advantage of AI‐guided 3D printing is its ability to customize solutions for each patient's unique facial structure and tissue characteristics. Algorithms can analyze both 2D and 3D data to design implants tailored to a patient's anatomy. For example, platforms like FITme's FACE ON system utilize AI to convert patient scans into surgical‐grade 3D models for cheek or jawline augmentation. Such systems facilitate preoperative visualization, allowing patients to preview expected results and encouraging collaborative decision‐making. While the potential is promising, peer‐reviewed validation of these technologies is still limited and should be approached with caution.

Beyond aesthetic applications, AI‐guided 3D printing is also valuable in reconstructive dermatology, particularly for post‐trauma and congenital deformity corrections. Soft tissue restoration using customized, bioresorbable materials such as polycaprolactone (PCL) provides a biocompatible alternative to traditional implants. These materials naturally degrade over time, enabling interim solutions and adaptive re‐treatments without permanent structural change. AI can assist in selecting appropriate materials and modeling patient‐specific degradation profiles, optimizing both functionality and aesthetic longevity [4].

Additionally, AI facilitates provider workflows by automating implant design, improving surgical planning, and reducing procedural times, which enhances overall efficiency. For instance, rapid prototyping of facial implants may minimize intraoperative adjustments, shorten anesthesia duration, and increase surgical precision.

However, the convergence of these technologies raises regulatory and ethical concerns. AI systems require large datasets containing sensitive biometric and medical information. The lack of robust frameworks regarding data privacy, algorithmic transparency, and validation poses significant challenges. Currently, the US FDA has issued general guidance for AI/ML‐based software as a medical device (SaMD), but specific pathways for AI‐designed, patient‐matched 3D‐printed implants are still being developed. Similarly, the European CE marking system does not yet provide detailed criteria for AI‐influenced custom manufacturing. Furthermore, clinical guidelines to govern the use of AI‐generated implants in aesthetic dermatology remain limited [5].

Algorithmic bias and limited model generalizability also warrant caution. AI tools trained primarily on homogeneous datasets may not perform well in diverse populations, potentially affecting outcome predictability. Issues such as overfitting and the lack of external validation further raise questions about reliability. Additionally, financial costs and the need for specialized training may hinder broader adoption.

To illustrate its clinical potential, consider a hypothetical case: a 35‐year‐old woman with post‐traumatic facial asymmetry seeks non‐surgical correction. Through AI‐derived facial symmetry analysis and 3D modeling, a patient‐specific filler mold is created using absorbable PCL. This approach not only restores facial contour with minimal downtime but also allows for future adjustments based on predicted degradation patterns, all visualized preoperatively through augmented reality simulation.

In conclusion, AI‐guided 3D printing presents a customizable, efficient, and patient‐centered approach in both aesthetic and reconstructive dermatology. While the potential is significant, success relies on scientific validation, ethical oversight, and regulatory alignment. With careful integration, these technologies can help shape the next era of dermatologic care.

## Disclosure

We confirm that the manuscript has been read and approved by all the authors, that the requirements for authorship as stated earlier in this document have been met and that each author believes that the manuscript represents honest work.

## Consent

Informed consent is unnecessary for this work.

## Conflicts of Interest

The authors declare no conflicts of interest.

## Data Availability

Data sharing not applicable to this article as no datasets were generated or analysed during the current study.

## References

[1] M. J. Zahid , P. Mavani , W. A. Awuah , et al., “Sculpting the Future: A Narrative Review of 3D Printing in Plastic Surgery and Prosthetic Devices,” Health Science Reports 7, no. 6 (2024): e2205. 38915353 10.1002/hsr2.2205 PMC11194296

[2] M. Fortune‐Ely , M. Achanta , and M. S. H. Song , “The Future of Artificial Intelligence in Facial Plastic Surgery,” JPRAS Open 39 (2024): 89–92. 38186379 10.1016/j.jpra.2023.11.016 PMC10770469

[3] A. Sriwastwa , P. Ravi , A. Emmert , et al., “Generative AI for Medical 3D Printing: A Comparison of ChatGPT Outputs to Reference Standard Education,” 3D Printing in Medicine 9, no. 1 (2023): 21, 10.1186/s41205-023-00186-8. 37525019 PMC10391950

[4] J. Parthasarathy , “3D Modeling, Custom Implants and Its Future Perspectives in Craniofacial Surgery,” Annals of Maxillofacial Surgery 4, no. 1 (2014): 9–18. 24987592 10.4103/2231-0746.133065 PMC4073471

[5] A. S. Deane and K. T. Byers , “A Review of the Ethical Considerations for the Use of 3D Printed Materials in Medical and Allied Health Education and a Proposed Collective Path Forward,” Anatomical Sciences Education 17, no. 6 (2024): 1164–1173. 39001638 10.1002/ase.2483

